# The effect of different teaching models on improving nursing humanistic care competencies: a network meta-analysis

**DOI:** 10.3389/fgene.2025.1602615

**Published:** 2025-07-09

**Authors:** Xiao Meixia, Xiong Qin, Jiang Rong, Li Lingxia, Fu Wenhao

**Affiliations:** ^1^ The First Affiliated Hospital of Nanchang University, Intensive Care Unit, Nanchang, Jiangxi, China; ^2^ Nanchang People’s Hospital, Operating Room, Nanchang, Jiangxi, China

**Keywords:** humanistic care, teaching modes, nurses, network meta-analysis, medical education

## Abstract

**Objectives:**

To evaluate the effectiveness of 13 teaching modes in improving nurses’ humanistic care ability using a Bayesian network meta-analysis.

**Methods:**

A computer-based search was conducted in PubMed, Web of Science, Cochrane Library, Embase, CBM, CNKI, WanFang, and VIP databases from their inception until 20 December 2024. Two researchers independently screened the literature, extracted data, and assessed the included studies using the bias risk assessment tool recommended by the Cochrane Handbook for Systematic Reviews of Interventions 5.1.0. The heterogeneity test was performed using RevMan 5.3 software, while the network meta-analysis and publication bias test were conducted using Stata 15.0 software.

**Results:**

A total of 21 studies involving 13 teaching modes (12 innovative teaching modes and Traditional Teaching Mode) and a combined sample size of 878 participants were included. All included studies were rated as either A or B in quality evaluation. Compared with the traditional teaching mode, the other 12 innovative teaching modes were more effective in enhancing nurses’ humanistic care ability, with statistically significant differences (P < 0.05). Among the 12 innovative teaching modes included, the “Peer Sister Teaching Mode” had superior teaching effectiveness compared to the other teaching modes. The Lasswell Teaching Mode was more effective than the Carolina Teaching Mode, Micro-video Teaching Mode, and Balint Teaching Mode. Additionally, the ADDIE Teaching Mode was more effective than the Carolina Teaching Mode and Micro-video Teaching Mode (P < 0.05). The cumulative ranking probability plot indicated that the “Learning from Sisters” teaching mode was the best for improving nurses’ humanistic care ability, with a probability of 91.7%.

**Conclusion:**

The “Peer Teaching Mode”, based on its peer - assisted and collaborative - learning approach, shows the most advantages in the network meta - analysis results. It is recommended that nursing management choose the “Peer Teaching Mode” as the best training strategy to enhance nurses’ humanistic care ability.

## 1 Introduction

In the rapidly evolving medical industry today, nurses play an indispensable role in the healthcare team. Their professional expertise and humanistic care abilities have a profound impact on patients’ physical and mental health, as well as the overall quality of medical services (Shuaifang and Zheng, 2024). Nursing humanistic care, centered on the patient, focuses on various aspects of patients’ needs, including physical, psychological, and social aspects. This holistic approach helps patients alleviate the physical and mental stress caused by illnesses, thereby boosting their confidence and courage to overcome diseases (Watson, 2011). However, there are still numerous deficiencies in nurses’ humanistic care abilities (Chunyan et al., 2023). On one hand, some nurses have an insufficient understanding of humanistic care, equating it simply with daily inquiries about patients’ wellbeing. This usually prevents them from fully grasping the essence and value of humanistic care, resulting in superficial and ineffective nursing behaviors in practical nursing work. On the other hand, nurses face numerous challenges in implementing humanistic care, such as heavy workloads, tense nurse-patient relationships, and complex clinical environments. These challenges make it difficult for nurses to fully consider patients’ psychological and social needs while managing their physical illnesses, leading to inadequate implementation of humanistic care. The traditional teaching model has overly emphasized the imparting of professional knowledge and skills, neglecting the systematic cultivation of students’ humanistic care awareness and abilities. Consequently, when nurses enter the workforce and encounter complex humanistic care needs, they often feel inadequate (Jianmei et al., 2023; Yu, 2024). As the quality nursing service work continues to be promoted in depth, a series of important documents such as the “National Nursing Development Plan (2021–2025)” (National Nursing Career Development Plan, 2020) have also clearly stated the need to improve nurses’ humanistic care abilities, build harmonious nurse-patient relationships, and enhance patient satisfaction.

Currently, strategies to improve nurses’ humanistic care abilities include establishing a culture of humanistic care, optimizing management systems and processes, strengthening training and assessment, and introducing patient feedback mechanisms. However, the most widely used approach in clinical settings is still the teaching and training model. To enhance nurses’ humanistic care abilities, numerous researchers and educators have actively explored the application effects of different teaching models (Zhou et al., 2024; Yuan et al., 2024), aiming to find more effective training pathways. Various teaching and training models have been reported in clinical settings, including experiential teaching, narrative nursing, Problem-Based Learning (PBL) teaching mode, and blended learning, all demonstrating positive effects on improving nurses’ humanistic care abilities (Wu, 2022). However, despite the diverse range of clinically reported teaching models with their unique characteristics, there is no unified consensus or standardization, limiting their widespread promotion (Chen et al., 2022; Ji et al., 2024; Zhan, 2023).

This study aims to conduct a systematic review and network meta-analysis of randomized controlled trials examining different humanistic care teaching models. By comparing the effects of various teaching models on enhancing humanistic care abilities and systematically evaluating their strengths and characteristics through subgroup analyses, we hope to provide valuable references for nursing educators. This, in turn, can facilitate domestic nursing education reform, promote the comprehensive improvement of nurses’ humanistic care abilities, and ultimately enable the provision of higher-quality and more humane nursing services to patients.

## 2 Materials and methods

The design and implementation plan of this study follows the Preferred Reporting Items for Systematic Reviews and Meta-Analyses (PRISMA) guidelines (Moher et al., 2009). This study has been registered on Prospero with the registration number CRD42025637832.

### 2.1 Search strategy

A comprehensive search was conducted in the Chinese Biomedical Literature Database, CNKI, Wanfang Database, VIP Chinese Science and Technology Journal Full-text Database, Cochrane Library, PubMed, Web of Science, and Wiley Online Library to identify studies on training methods for improving nurses’ humanistic care abilities. The search covered the period from the establishment of the databases to May 2024. Additionally, reference lists of included studies were traced. The search strategy combined subject headings and free-text terms. Chinese search terms included: (“nurses” OR “nursing staff” OR “standardized training nurses” OR “newly recruited nurses”) AND (“humanistic care” OR “caring ability” OR “humanistic care ability” OR “humanistic quality” OR “humanistic care education” OR “humanistic spirit”) AND (“random” OR “randomized controlled trial”). English search terms were: (nursing OR nurse) AND (care ability OR humanistic care OR humanistic care education OR caring behaviour) AND (randomized controlled trail OR RCT). The search strategy is exemplified by the Pubmed database (Table 1).

**TABLE 1:** The specific search strategy of PubMed database.

N	Search items
1	nurses [MESH]
2	(Nurse)OR (Nursing Personnel) OR (Registered Nurses)OR (Nurse, Registered) OR (Nurses, Registered)OR (Registered Nurse)
3	#1 OR #2
4	Humane care [MESH]
5	(humanistic care)OR (Humane care)OR (compassionate care)OR (care ability)OR (humanistic care education)OR (caring behaviour)
6	#4 OR #5
7	Educational Model [MESH]
8	(Educational Model)OR (Instructional Model)OR (Model, Instructional)OR (Educational Models)OR (Educational Model)OR (Model, Educational)
9	#7 OR #8
10	#3 AND #6 AND #9

### 2.2 Inclusion and exclusion criteria

Inclusion Criteria: (1) Study type: Randomized controlled trials (RCTs) published domestically and internationally; (2) Participants: Nursing staff aged 18 years or older; (3) Intervention: The experimental group received teaching modes such as PBL, experiential, narrative nursing, Balint group training, Micro-video teaching, or Mixed teaching, while the control group followed traditional teaching methods; (4) Research tools: Assessment of humanistic care abilities using scales such as the Humanistic Care Ability Evaluation Scale or the Humanistic Professional Ability Evaluation Scale; (5) Outcome measures: Humanistic care ability, humanistic professional ability, and quality of humanistic care. Exclusion Criteria: (1) Studies with inaccessible or missing data, and no response from the original author after three consecutive email contacts; (2) Duplicate publications; (3) Literature with a quality grade of C; (4) Non-Chinese or non-English publications.

### 2.3 Literature screening and data extraction

Based on the research objectives and inclusion criteria, two researchers independently screened the titles and abstracts of the retrieved literature. Full texts of the initially included studies were read, and the following data were extracted: first author, publication year, participants’ age, number of cases, intervention measures, intervention duration, and evaluation indicators. When complete data extraction was unclear or difficult for the two researchers, the original author was contacted for complete data. If the original data could not be obtained after three consecutive weeks of email contact, the study was defined as missing data. Additionally, any differences in screening opinions between the two researchers were resolved through scientific meetings initiated by the principal investigator.

### 2.4 Bias risk evaluation

Two researchers independently evaluated the bias risk of the included literature using the RCT bias risk assessment tool recommended by the Cochrane Handbook for Systematic Reviews of Interventions 5.1.0. The evaluation covered seven aspects, including random sequence generation, allocation concealment, blinding of participants or interveners, blinding of outcome assessors, completeness of data, selective reporting, and other bias risks. Each item was evaluated as “low bias risk,” “high bias risk,” or “unclear” and presented through a risk of bias graph using corresponding “green,” “red,” and “yellow” markings. The literature quality grade was set as “A” if it fully met the evaluation criteria, “B” if it partially met the criteria, and “C” if it did not meet the criteria at all. Only two studies fully met the evaluation criteria and were rated as A in terms of literature quality. The other studies were rated as B due to the failure to use appropriate randomization methods or implement blinding. Disagreements in bias risk evaluation were resolved through scientific meetings initiated by the principal investigator.

### 2.5 Statistical methods

RevMan 5.3 software was used for heterogeneity testing, traditional meta-analysis, and publication bias testing. For continuous variables, the combined effect size was represented by the standardized mean difference (SMD) and 95% confidence interval (95% CI). The Q test was used to determine whether there was heterogeneity among studies, and the I^2^ statistic was used to assess the magnitude of heterogeneity. If I^2^ < 50% and P > 0.10, a fixed-effects model was used for analysis; if I^2^ ≥ 50% and P ≤ 0.10, a random-effects model was used for analysis. Addis 16.6 software was used to draw the network evidence diagram to analyze whether the included studies met the prerequisites for network meta-analysis. Data were analyzed and processed based on the Bayesian framework and Markov chain Monte Carlo methods. In Addis 16.6 software, four Markov chains were set with an initial value of 2.5, 20,000 annealing iterations, 50,000 simulation iterations, and 10 thinning iterations. The potential scale reduction factor (PSRF) was used to assess model convergence. When the PSRF tended to 1, it indicated that the model had converged satisfactorily, and the consistency model analysis conclusion had a high degree of credibility. When the evidence network had a closed loop, the consistency between direct and indirect comparisons was judged by the node split value. When P < 0.05, it could be considered that the inconsistency was significant. The surface under the cumulative ranking (SUCRA) was used to show the probability of each intervention being the best intervention. The superiority of interventions was ranked according to the SUCRA values.

## 3 Results

### 3.1 Literature screening process and results

**FIGURE 1:**
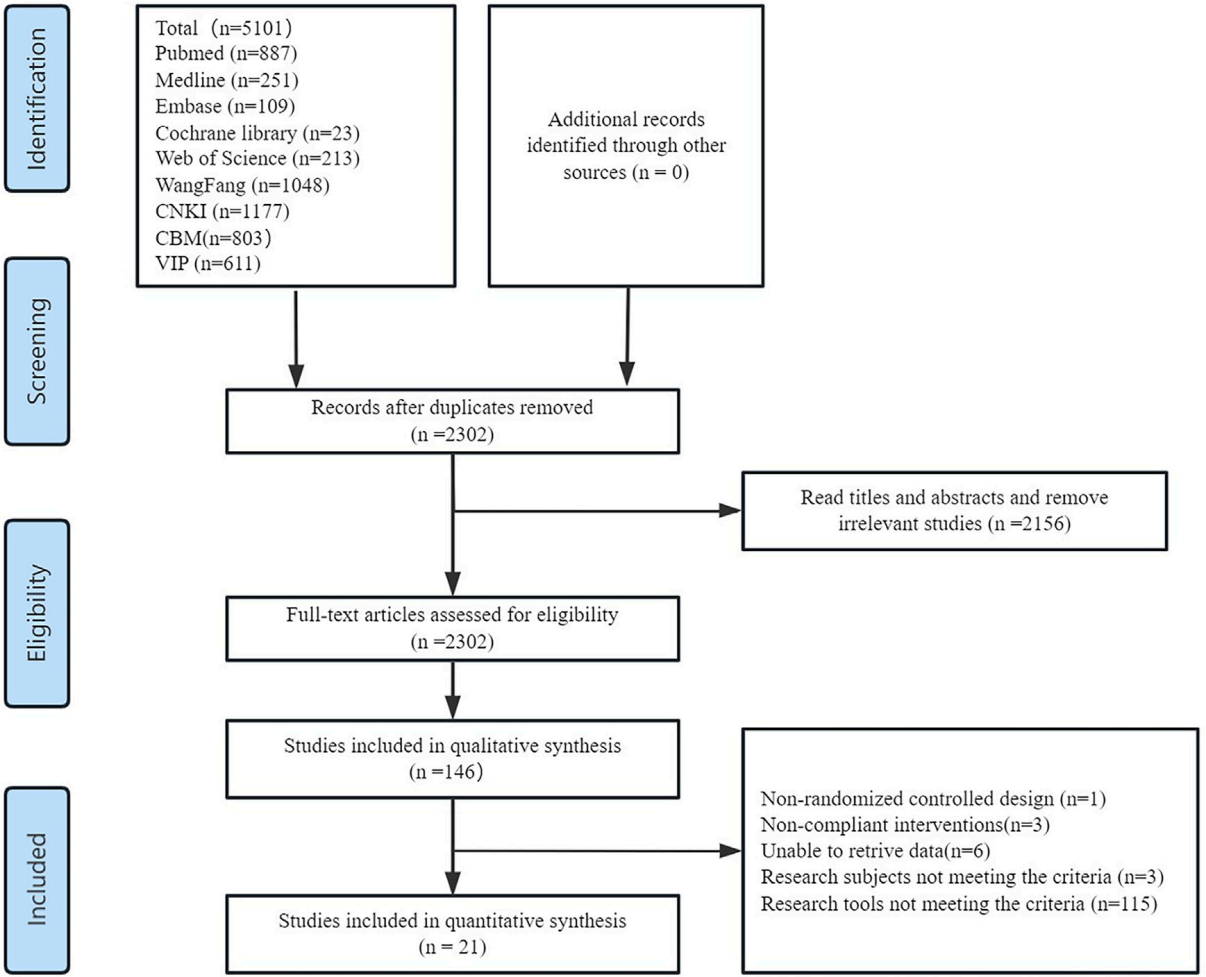
Literature screening flowchart and results.

The initial search yielded 1,483 English articles and 3,618 Chinese articles. Using Note Express 3.2 software, 1799 duplicate articles were removed. After title and abstract screening, 146 articles were selected for full-text review. After downloading and reading the full texts of the remaining articles, 115 studies with unsuitable research tools or additional institutional interventions,3 studies with non-clinical nurse participants, 6 studies with incomplete data, and 1 non-randomized controlled study were excluded. Finally, 21 RCTs were included (Figure 1).

### 3.2 Basic characteristics of included literature

The 21 studies (Chen et al., 2022; Gu et al., 2022; Yan et al., 2022; Wang and Zhang, 2022; Hu et al., 2021; Zhang et al., 2021; Gao, 2021; Jiang et al., 2021; Peng et al., 2021; Zheng and Sun, 2020; Lv et al., 2020; Liu, 2020; Zhang, 2020; Yu et al., 2019; Chen et al., 2019; Wang et al., 2018; Song, 2018; Shi and Wang, 2018; Wang, 2017; Wang et al., 2020; Su, 2019) included in this review are all randomized controlled trials comparing different teaching modes with Traditional Teaching Mode. A total of 878 nurses were involved, with the majority of participants aged between 20 and 30 years old. The intervention periods varied significantly, ranging from 6 weeks to 24 months. Collectively, these studies examined 13 teaching modes and utilized 4 different assessment tools for evaluating humanistic care abilities. The teaching modes encompassed were Experiential Teaching Mode, Narrative Nursing Teaching Mode, Balint Group Teaching Mode, Micro-video Teaching Mode, Blended Teaching Mode, Reflective Teaching Mode, Peer Teaching Mode, PBL Teaching Mode, Step-by-Step Teaching Mode, Carolina Teaching Mode, Analysis-Design-Development-Implementation-Evaluation (ADDIE) Teaching Mode, Lasswell Teaching Mode, and Traditional Teaching Mode. The 4 assessment tools for humanistic care abilities included the Caring Ability Inventory (CAI) (Hung, 2020), Humanistic Professional Competence Evaluation Scale (Yan, 2016), Liu Yujing’s Humanistic Care Quality Evaluation Scale (Liu, 2011), and Huang Yibing’s Humanistic Care Ability Evaluation Scale (Huang, 2007) (Table 2). CAI mainly assesses nurses’ implementation ability in providing emotional support, empathetic understanding, and personalized care to patients in caring practice, focusing on the skill dimension of caring behaviors. HPA focuses on evaluating nurses’ comprehensive professional quality and level of action transformation in clinical practice, which includes the integration of professional knowledge, ethical decision-making, communication skills, and the concept of humanistic care. HCQ centers on nurses’ values, moral integrity, and intrinsic caring qualities, measuring their empathy, sense of responsibility, and depth of understanding of the dignity of life. HCA systematically evaluates the overall performance of nursing staff in caring practice through multidimensional indicators (such as cognition, emotion, and behavior), covering awareness of caring, practical effectiveness, and the ability for continuous improvement.

**TABLE 2:** Basic characteristics of the included literature.

Study	Published (Y)	Sample size (N)	Age (years)	Intervention	Number of sessions	Evaluation tool
(Gu et al., 2022)	2022	Control group (36) Experimental group (36)	22.2 ± 0.43 22.3 ± 0.31	Traditional Teaching Mode PBL Teaching Mode	3 months	CAI
(Yan et al. 2022)	2022	Control group (93) Experimental group (98)	NR	Traditional Teaching Mode Experiential Teaching Mode	3 months	HPA
(Wang and Zhang, 2022)	2022	Control group (21) Experimental group (21)	23.35 ± 3.12 23.74 ± 2.98	Traditional Teaching Mode Narrative Nursing Teaching Mode	3 months	CAI
(Hung, 2020)	2022	Control group (29) Experimental group (30)	23.16 ± 1.89 22.84 ± 2.05	Traditional Teaching Mode Experiential Teaching Mode	2 months	CAI
(Hu et al., 2021)	2021	Control group (55) Experimental group (36)	NR	Traditional Teaching Mode Balint Group Teaching Mode	12 months	HCA
(Zhang et al., 2021)	2021	Control group (25) Experimental group (25)	21.36 ± 0.81 21.08 ± 0.7	Traditional Teaching Mode Micro-video Teaching Mode	6 weeks	CAI
(Gao, 2021)	2021	Control group (20) Experimental group (20)	26.85 ± 3.50 29.05 ± 5.81	Traditional Teaching Mode Carolina Teaching Mode	3 months	CAI
(Jiang et al., 2021)	2021	Control group (35) Experimental group (35)	30.80 ± 7.45 28.39 ± 6.30	Traditional Teaching Mode Blended Teaching Mode	6 months	HCQ
(Peng et al., 2021)	2021	Control group (62) Experimental group (62)	20.98 ± 4.16 21.41 ± 3.24	Traditional Teaching Mode PBL Teaching Mode	2 months	CAI
(Zheng and Sun, 2020)	2020	Control group (15) Experimental group (15)	20.6 ± 3.3 20.3 ± 3.6	Traditional Teaching Mode Blended Teaching Mode	6 months	CAI
(Lv et al., 2020)	2020	Control group (80) Experimental group (80)	NR	Traditional Teaching Mode Balint Group Teaching Mode	6 months	HCQ
(Liu, 2020)	2020	Control group (18) Experimental group (18)	NR	Traditional Teaching Mode Narrative Nursing Teaching Mode	3 months	CAI
(Zhang, 2020)	2020	Control group (24) Experimental group (24)	NR	Traditional Teaching Mode Reflective Teaching Mode	NR	HCA
(Wang et al., 2020)	2020	Control group (32) Experimental group (37)	22.35 ± 1.37 22.51 ± 1.62	Traditional Teaching Mode Carolina Teaching Mode	1 month	HCQ
(Yu et al., 2019)	2019	Control group (93) Experimental group (92)	21.19 ± 2.01 21.36 ± 2.09	Traditional Teaching Mode Peer Teaching Mode	24 months	HCQ
(Chen et al., 2019)	2019	Control group (25) Experimental group (25)	21.87 ± 1.89 21.54 ± 1.65	Traditional Teaching Mode Blended Teaching Mode	NR	HCA
(Su, 2019)	2019	Control group (23) Experimental group (23)	NR	Traditional Teaching Mode Step-by-Step Teaching Mode	NR	HCA
(Wang et al., 2018)	2018	Control group (45) Experimental group (45)	24.43 ± 1.5 24.12 ± 1.8	Traditional Teaching Mode Experiential Teaching Mode	NR	CAI
(Song, 2018)	2018	Control group (65) Experimental group (69)	NR	Traditional Teaching Mode ADDIE Teaching Mode	6 weeks	HCA
(Shi and Wang, 2018)	2018	Control group (30) Experimental group (30)	23.12 + 1.08 23.05 + 1.2	Traditional Teaching Mode Experiential Teaching Mode	NR	CAI
(Wang, 2017)	2017	Control group (25) Experimental group (25)	NR	Traditional Teaching Mode Lasswell Teaching Mode	3 months	CAI

Y:years; N: number; CAI: caring ability inventory; HPA:humanities practice ability; HCA:Humanistic Caring Ability; HCQ: humanistic care quality.

### 3.3 Results of quality assessment

Of the 21 included studies, 2 studies were grade A literature (Song, 2018; Li et al., 2020) and 19 studies were grade B literature (Chen et al., 2022; Gu et al., 2022; Yan et al., 2022; Wang and Zhang, 2022; Hu et al., 2021; Zhang et al., 2021; Gao, 2021; Jiang et al., 2021; Peng et al., 2021; Zheng and Sun, 2020; Lv et al., 2020; Liu, 2020; Zhang, 2020; Yu et al., 2019; Chen et al., 2019; Song, 2018; Wang, 2017; Su, 2019). Seventeen of these studies (Chen et al., 2022; Gu et al., 2022; Wang and Zhang, 2022; Hu et al., 2021; Zhang et al., 2021; Gao, 2021; Jiang et al., 2021; Zheng and Sun, 2020; Lv et al., 2020; Liu, 2020; Zhang, 2020; Yu et al., 2019; Chen et al., 2019; Wang et al., 2018; Shi and Wang, 2018; Wang, 2017; Wang et al., 2020) reported specific methods of random sequence generation, mainly lottery and random number table methods; six studies (Chen et al., 2022; Hu et al., 2021; Yu et al., 2019; Wang et al., 2018; Song, 2018; Wang et al., 2020) mentioned whether or not allocation was hidden, but none of them mentioned what form of blinding was used; and judging the loss of visits from the number of grouped cases and the number of reported cases of results, the 20 studies had no lost visits or sample drop-outs, and only one study (Chen et al., 2019) reported lost visits, with good data completeness (Table 3).

**TABLE 3:** Offset risk results for the included literature.

Study	Random sequence generation (selection bias)	Assignment hiding	Masking		Integrity of data results	Selective reporting of results	Other biases	Quality level
			Research subjects or interveners	Outcome measurers				
(Gu et al., 2022)	Low	NR	High	High	Low	Low	Low	B
(Yan et al., 2022)	NR	NR	High	High	Low	Low	Low	B
(Wang and Zhang, 2022)	Low	NR	High	Low	Low	Low	NR	B
(Hung, 2020)	Low	Low	High	Low	Low	Low	Low	B
(Hu et al., 2021)	Low	Low	High	Low	Low	Low	Low	B
(Zhang et al., 2021)	Low	NR	High	Low	Low	Low	Low	B
(Gao, 2021)	High	High	Low	Low	Low	Low	Low	B
(Jiang et al., 2021)	Low	High	High	Low	Low	Low	Low	B
(Peng et al., 2021)	NR	High	High	NR	Low	Low	Low	B
(Zheng and Sun, 2020)	Low	NR	High	NR	Low	Low	Low	B
(Lv et al., 2020)	Low	NR	High	Low	Low	Low	Low	B
(Liu, 2020)	Low	High	High	Low	Low	Low	Low	B
(Zhang, 2020)	Low	High	High	High	Low	Low	Low	B
(Yu et al., 2019)	Low	Low	Low	High	Low	Low	Low	B
(Chen et al., 2019)	Low	NR	High	High	Low	Low	Low	B
(Su, 2019)	NR	High	High	Low	Low	Low	Low	B
(Wang et al., 2018)	Low	Low	Low	Low	Low	Low	Low	A
(Song, 2018)	NR	Low	Low	Low	Low	Low	Low	B
(Shi and Wang, 2018)	Low	High	High	Low	Low	Low	Low	B
(Wang, 2017)	Low	High	High	Low	Low	Low	Low	B
(Wang et al., 2020)	Low	Low	Low	Low	Low	Low	Low	A

### 3.4 Results of heterogeneity test

The 21 included studies involved a total of 12 comparison schemes. Under the same comparison schemes, the heterogeneity test results (comparing experiential teaching mode with Traditional Teaching Mode, Narrative Nursing Teaching Mode with Traditional Teaching Mode, Balint Teaching mode withTraditional Teaching Mode, Micro-video Teaching Mode with Traditional Teaching Mode, Blended Teaching Mode with Traditional Teaching Mode, Reflective Teaching Mode with Traditional Teaching Mode, Peer Teaching Mode with Traditional Teaching Mode, PBL Teaching Mode with Traditional Teaching Mode, Stepped Teaching Mode with Traditional Teaching Mode, Carolina Teaching Mode with Traditional Teaching Mode, ADDIE Teaching Mode with Traditional Teaching Mode, and Lasswell Teaching Mode with Traditional Teaching Mode) showed that *I* ^2^ > 50% and *P* < 0.05. Therefore, we adopted a random effects model to conduct a traditional meta-analysis of the 21 studies and used SMD and 95% CI values to represent the pooled effect size.

### 3.5 Network structure diagram

**FIGURE 2:**
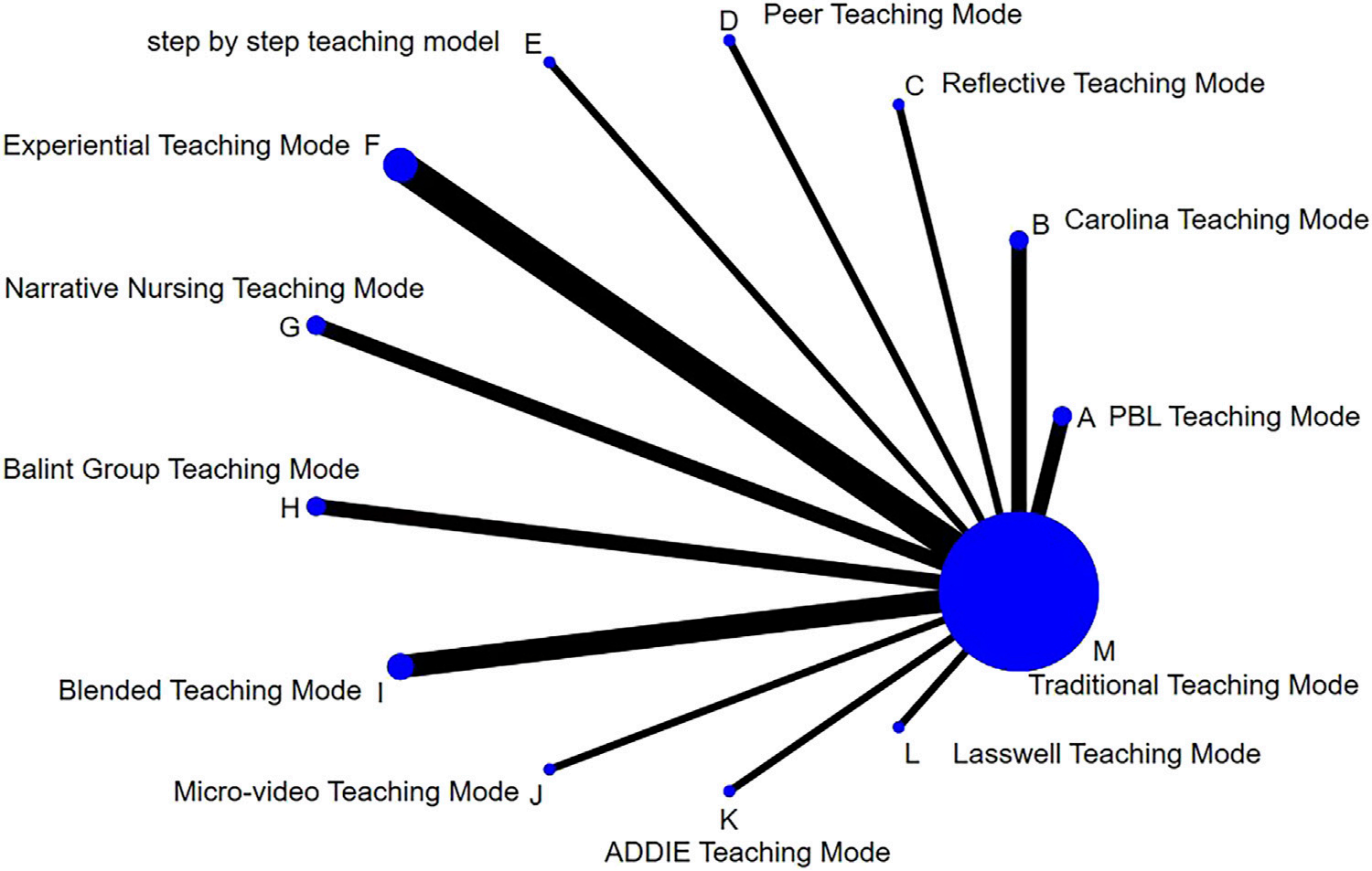
Comparison of bayesian network meta-analysis of interventions in different teaching modes.

All 21 included studies were two-arm randomized controlled trials designed to compare the superiority of a single teaching mode with the Traditional Teaching Mode. In this study, the Traditional Teaching Mode had the largest proportion of sample size, followed by the Experiential Teaching Mode and the Blended Teaching Mode. These two teaching modes also accounted for the largest proportion of studies. Finally, in the network structure diagram, a network evidence map was formed based on direct comparison data: the most studies compared Experiential Teaching Mode with Traditional Teaching Mode, followed by Blended Teaching Mode, Narrative Nursing Teaching Mode, Balint Group Teaching Mode, Carolina Teaching Mode, PBL Teaching Mode, and lastly, Step-by-Step Teaching Mode, Peer Teaching Mode, Reflective Teaching Mode, Micro-Video Teaching Mode, and ADDIE Teaching Mode (Figure 2).

### 3.6 Meta-analysis results

**FIGURE 3:**
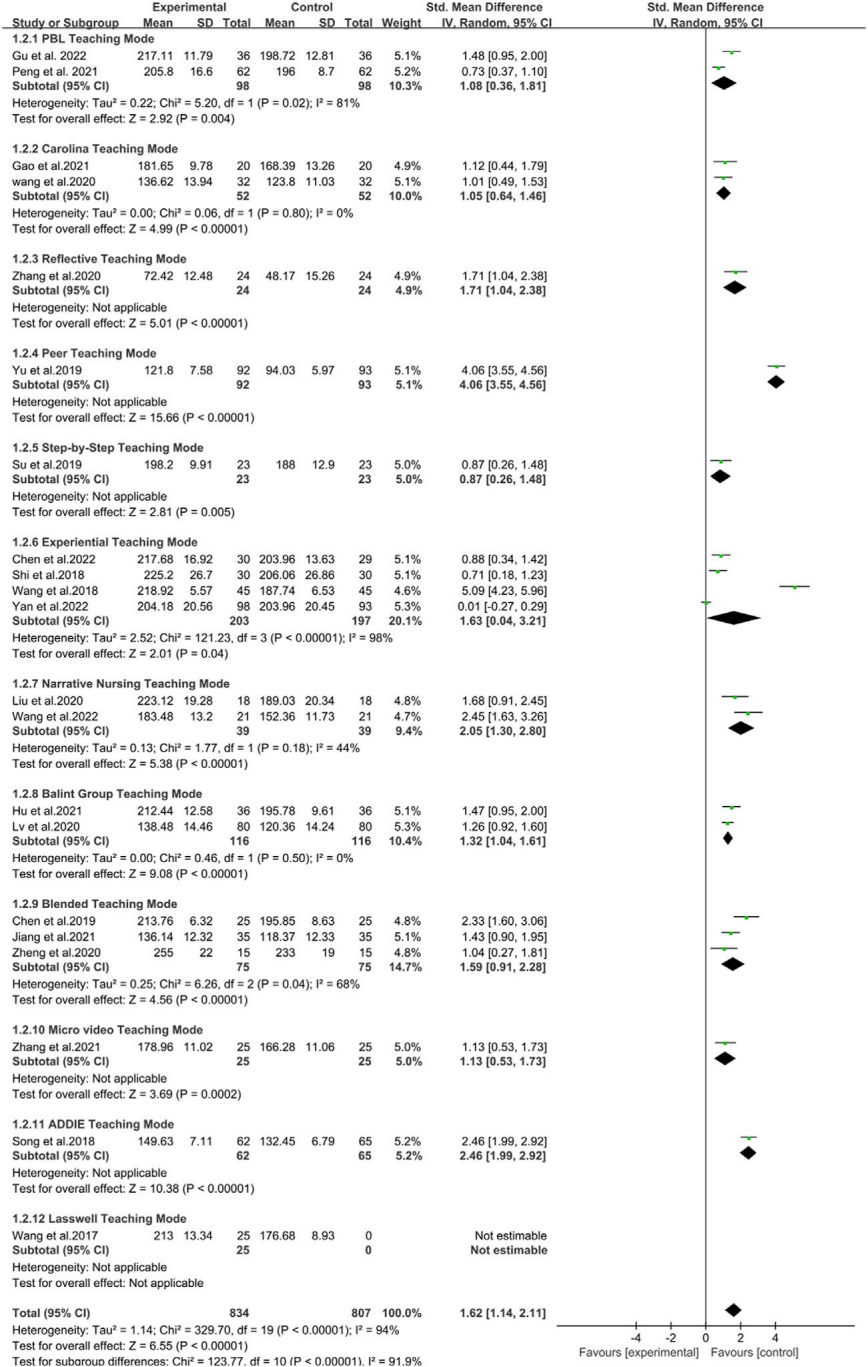
Forest plot of the impact of different teaching modes on enhancing nurses’ humanistic care abilities.

The results of the traditional meta-analysis indicate that, compared to Traditional Teaching Mode, novel teaching modes are more effective in enhancing the humanistic care abilities of participants. Specifically, among the 12 teaching modes examined, all demonstrated varying degrees of superiority over traditional modes in improving the humanistic care capabilities of nursing professionals. The PBL Teaching Mode was found to be superior to the Traditional Teaching Mode [SMD = 1.08, 95%CI (0.36, 1.81), *P* < 0.05]. Similarly, the Carolina Teaching Mode [SMD = 1.05, 95%CI(0.64, 1.46), *P* < 0.05], Reflective Teaching Mode [SMD = 1.71, 95%CI(1.04, 2.38), *P* < 0.05], Peer Teaching Mode [SMD = 4.06, 95%CI(3.55, 4.56), *P* < 0.05], Stepped Teaching Mode [SMD = 0.87, 95%CI(0.26, 1.48), P < 0.05], Experiential Teaching Mode [SMD = 1.72, 95%CI(0.27, 3.17), *P* < 0.05],Narrative Nursing Teaching Mode [SMD = 2.05, 95%CI(1.30, 2.80), *P* < 0.05], Balint Group Teaching Mode [SMD = 1.32, 95%CI(1.04, 1.61), *P <* 0.05], Blended Teaching Mode [SMD = 1.59, 95%CI(0.91, 2.28), *P* < 0.05], Micro-Video Teaching Mode [SMD = 1.13, 95%CI(0.53, 1.73), *P* < 0.05], ADDEI Teaching Mode [SMD = 2.46, 95%CI(1.99, 2.92), *P* < 0.05], and the Lasswell Teaching Mode [SMD = 3.15, 95%CI(2.30, 4.00), *P* < 0.05] were all found to be significantly better than the traditional teaching mode (Figure 3). Moreover, we can intuitively see from the figure that the combined effect sizes demonstrated by the Peer Teaching Mode and Lasswell Teaching Modes (Peer Teaching Mode: SMD = 4.06 [3.55, 4.56], Lasswell Teaching Mode: SMD = 3.15 [2.30, 4.00]) are superior to other teaching modes in enhancing the humanistic care abilities of nursing personnel, showing a stronger advantage in this regard (Figure 3). Additionally, in Figure 3, the innovative teaching modes Experiential Teaching Mode and Blended Teaching Mode both exhibited high heterogeneity (*I* ^2^ > 80%). Our research team analyzed the intervention content and evaluation methods of these RCTs and found that all studies used the same type of nursing humanistic care evaluation scale. We considered that the source of high heterogeneity might be the different educational levels of the units in which the studies were conducted or the different total durations of education that were not reported in detail in the studies.

**FIGURE 4:**
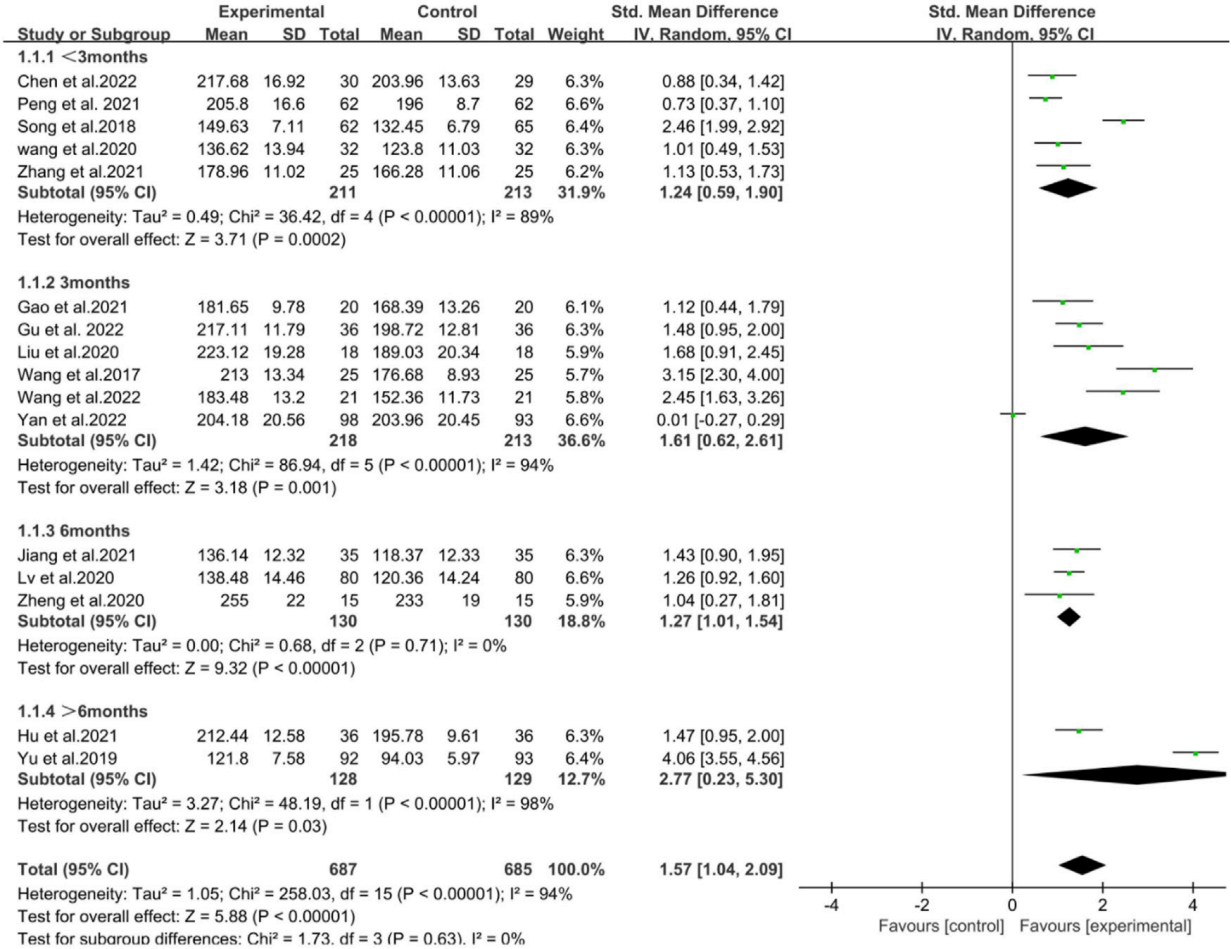
Forest plot of the impact of innovative teaching models with different training durations on enhancing nurses’ humanistic care abilities.

Additionally, the subgroup analysis based on the duration of the training cycle found that compared with the Traditional Teaching Mode, the innovative education model with different training durations all effectively enhanced the humanistic care ability of nursing staff. However, when the training cycle exceeded 6 months, the training outcomes were more optimal (<3 months [SMD = 1.24, 95% CI (0.59, 1.90), *P* < 0.05]; 3 months [SMD = 1.61, 95% CI (0.62, 2.61), *P* < 0.05]; 6 months [SMD = 1.27, 95% CI (1.01, 1.54), *P* < 0.05]; >6 months [SMD = 2.77, 95% CI (0.23, 5.30), *P* < 0.05]). Thus, it can be seen that the duration of training in the innovative education model of nursing humanistic care ability may have a certain impact on enhancing the humanistic care ability of nursing staff. Future further research should be based on a teaching cycle with a training duration exceeding 6 months to achieve more optimal research outcomes (Figure 4). In our forest plot, some studies exhibited high heterogeneity. Differences in sample size and variations in the content of the innovative educational interventions could both potentially lead to the emergence of heterogeneity. However, the results of the subgroup analysis found that the more likely source of the higher heterogeneity is the use of different training durations in the RCTs of various innovative teaching models.

The results of the network meta-analysis showed that various teaching modes were superior to Traditional Teaching Mode in terms of improving nurses’ humanistic care abilities (*P* < 0.05). Specifically, the Peer Teaching Mode proved to be more effective than other teaching methods (P < 0.05). The Lasswell Teaching Mode demonstrated superiority over the traditional teaching mode [SMD = 3.15, 95% CI (0.54, 5.75), *P* < 0.05], as well as over the Carolina Teaching Mode [SMD = 2.09, 95% CI (1.08, 5.25), *P* < 0.05], the Micro video Teaching Mode [SMD = 2.02, 95% CI (1.62, 5.65), *P* < 0.05], and the Balint Group Teaching Mode [SMD = 1.78, 95% CI (1.37, 4.93), *P* < 0.05]. Additionally, the ADDIE Teaching Mode was found to be superior to the Carolina Teaching Mode [SMD = 1.40, 95% CI (1.09, 4.48), *P* < 0.05], the Traditional Teaching Mode [SMD = 2.46, 95% CI (0.05, 4.96), *P* < 0.05], and the Micro-video Teaching Mode [SMD = 1.33, 95% CI (1.24, 4.89), *P* < 0.05] (Table 4). Additionally, it should be noted that the confidence intervals for some comparisons are relatively wide (e.g., K vs. G: CI -2.71–3.50), which may lead to unstable estimates. The possible reasons for this may come from several aspects. On the one hand, the small sample size causes the sampling distribution of the estimates to be more dispersed, resulting in wider confidence intervals. Future further research should increase the sample size on this basis to reduce the standard error and thus narrow the confidence intervals. On the other hand, the effect size of some studies is relatively small, and a larger sample size is needed to detect this effect. Similarly, when the sample size is small, wide confidence intervals are used to compensate for the detection of this effect.

**TABLE 4:** Network meta-analysis of the effectiveness of different teaching models in improving nurses’ humanistic care competency Levels [SMD(95%CI)].

措施	D	L	K	G	F	C	I	H	J	A	B	E	M
D	-^①^	-	-	-	-	-	-	-	-	-	-	-	-
L	0.91 (2.71,4.53)^①^	-	-	-	-	-	-	-	-	-	-	-	-
K	1.60 (1.95,5.15)^①^	0.69 (−2.93,4.31)	-	-	-	-	-	-	-	-	-	-	-
G	1.99 (1.12,5.11)^①^	1.09 (−2.10,4.27)	0.40 (−2.71,3.50)	-	-	-	-	-	-	-	-	-	-
F	2.35 (0.47,5.17)	1.44 (−1.46,4.34)	0.75 (−2.06,3.56)	0.35 (-1.87,2.58)	-	-	-	-	-	-	-	-	-
C	2.34 (1.24,5.93)^①^	1.44 (−2.21,5.09)	0.75 (-2.83,4.33)	0.35 (−2.79,3.49)	0.01 (−2.85,2.85)	-	-	-	-	-	-	-	-
I	2.46 (0.46,5.37)	1.55(-1.45,4.54)	0.86 (−2.05,3.77)	0.46 (−1.89,2.81)	0.11(-1.84,2.05)	0.11 (−2.84,3.06)	-	-	-	-	-	-	-
H	2.69 (0.38,5.77)^①^	1.78(1.37,4.93)^①^	1.09 (−1.97,4.16)	0.70 (−1.85,3.24)	0.34 (−1.83,2.52)	0.35 (−2.76,3.45)	0.23 (−2.07,2.54)	-	-	-	-	-	-
J	2.92 (0.65,6.50)^①^	2.02 (1.62,5.65)^①^	1.33 (1.24,4.89)^①^	0.93 (−2.20,4.06)	0.58 (−2.26,3.41)	0.58(-3.02,4.18)	0.47(-2.47,3.40)	0.23 (−2.86,3.33)	-	-	-	-	-
A	2.96 (0.11,6.02)^①^	2.05(-1.10,5.19)	1.36(-1.70,4.42)	0.96(-1.58,3.50)	0.61 (−1.56,2.77)	0.61 (−2.49,3.71)	0.50 (−1.80,2.79)	0.26(-2.23,2.76)	0.03 (−3.05,3.12)	-	-	-	-
B	2.99 (0.09,6.08)^①^	2.09 (1.08,5.25)^①^	1.40 (1.09,4.48)^①^	1.00 (−1.56,3.56)	0.65 (−1.55,2.84)	0.65 (−2.47,3.77)	0.54 (−1.79,2.86)	0.30(-2.22,2.82)	0.07 (−3.04,3.18)	0.04 (−2.47,2.55)	-	-	-
E	3.18 (0.39,6.76)^①^	2.28(-1.36,5.91)	1.59 (−1.98,5.15)	1.19 (−1.94,4.32)	0.83 (−2.00,3.67)	0.84 (−2.76,4.44)	0.73 (−2.21,3.66)	0.49 (−2.60,3.59)	0.26 (−3.33,3.85)	0.23(-2.86,3.31)	0.19(-2.92,3.30)	-	-
M	4.06 (1.54,6.57)^①^	3.15(0.54,5.75)^①^	2.46 (0.05,4.96)^①^	2.06(0.23,3.89)^①^	1.71 (0.44,2.97)^①^	1.71 (0.84,4.26)^①^	1.60 (0.12,3.07)^①^	1.36 (0.41,3.13)^①^	1.13 (1.41,3.67)^①^	1.10(0.66,2.86)^①^	1.06(0.73,2.85)^①^	0.87(1.67,3.41)^①^	-

A = PBL, Teaching Mode B = Carolina Teaching Mode C = Reflective Teaching Mode D = Peer Teaching Mode E = Step-by-Step Teaching Mode F = Experiential Teaching Mode G = Narrative Nursing Teaching Mode H = Balint Group Teaching Mode I = Blended Teaching Mode J = Micro video Teaching Mode K = ADDIE, Teaching Mode L = Lasswell Teaching Mode M = Traditional Teaching Mode。① Indicates *P* < 0.05; - Indicates no relevant data/duplicate data (not shown); SMD, standardized mean difference.

### 3.7 Ranking results

**FIGURE 5:**
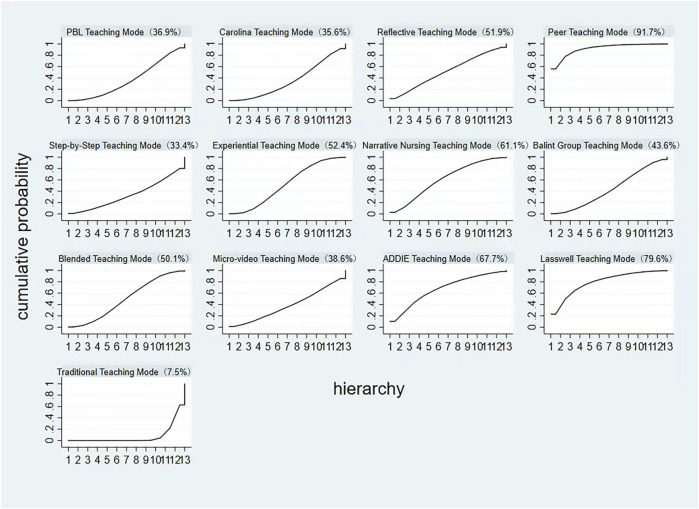
Probability ranking plot of the impact of different teaching modes on enhancing nurses’ humanistic care abilities.

The ranking probability graph also indicates the intervention effects of different teaching modes on improving nurses’ humanistic care abilities. The order of effectiveness from highest to lowest is as follows: Peer Teaching Mode (with a top-ranked intervention effect probability of 91.7%), Lasswell Teaching Mode (79.6% probability of being the most effective), ADDIE Teaching Mode (67.7% probability of being the most effective), Narrative Nursing Teaching Mode (61.1% probability of being the most effective), Experiential Teaching Mode (52.4% probability of being the most effective), Reflective Teaching Mode (51.9% probability of being the most effective), Blended Teaching Mode (50.1% probability of being the most effective), Balint Group Teaching Mode (43.6% probability of being the most effective), Micro-video Teaching Mode (38.6% probability of being the most effective), PBL Teaching Mode (36.9% probability of being the most effective), Carolina Teaching Mode (35.6% probability of being the most effective), Step-by-Step Teaching Mode (33.4% probability of being the most effective), and Traditional Teaching Mode (7.5% probability of being the most effective) (Figure 5).

### 3.8 Publication bias test

**FIGURE 6:**
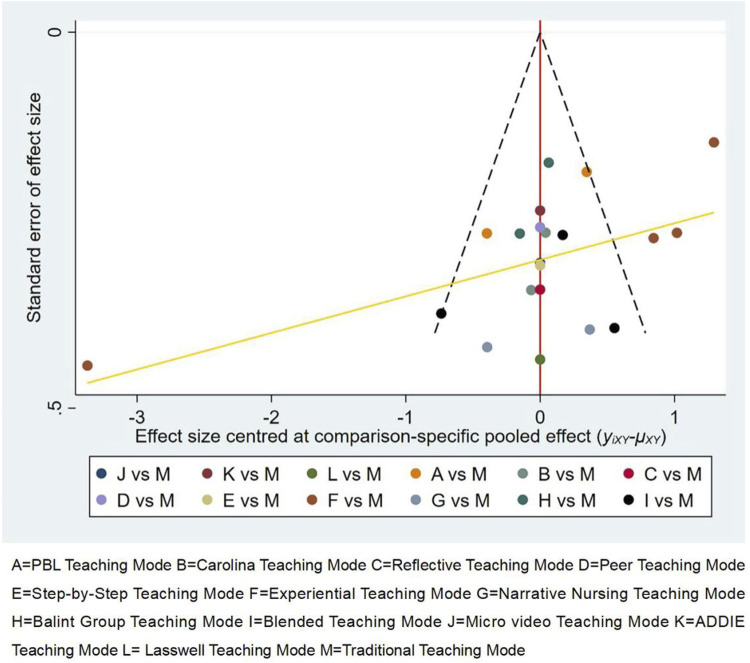
Funnel Plot of Adjusted Comparisons of Different Teaching Modes. A = PBL Teaching Mode B = Carolina Teaching Mode C = Reflective Teaching Mode D = Peer Teaching Mode E = Step-by-Step Teaching Mode F = Experiential Teaching Mode G = Narrative Nursing Teaching Mode H = Balint Group Teaching Mode I = Blended Teaching Mode J = Micro video Teaching Mode K = ADDIE Teaching Mode L = Lasswell Teaching Mode M = Traditional Teaching Mode.

A funnel plot was drawn to show the impact of different teaching modes on promoting nurses’ humanistic care ability (Figure 6). The Experiential Teaching Mode lay outside the funnel plot, indicating high heterogeneity. Other modes were symmetrically distributed around the X = 0 line within the confidence interval. The main causes are the small sample size and teaching - mode differences leading to a high I^2^ value, making the funnel plot centrally symmetric except for the Experiential Teaching Mode outside the confidence interval. As an emerging humanistic care training method, the Experiential Teaching Mode draws researchers’ interest in exploring its strengths, which can cause selection bias. Also, studies on this model need more resources. Due to limited resources, only a few well - resourced studies are conducted and published, which can’t represent all studies on this mode, thus causing bias.

## 4 Discussion

This study aimed to effectively enhance the humanistic care abilities of nurses through various teaching modalities. Utilizing a Bayesian network meta-analysis, we compared the efficacy of 12 novel teaching modalities with Traditional Teaching Mode in improving nurses’ humanistic care abilities. The findings revealed that all 12 novel teaching modalities surpassed the traditional approach. Furthermore, due to their distinct teaching characteristics, these modalities exhibited varying degrees of effectiveness. The ranking, from most to least effective, was as follows: Peer Teaching Mode, Lasswell Teaching Mode, ADDIE Teaching Mode, Narrative Nursing Teaching Mode, Experiential Teaching Mode, Reflective Teaching Mode, Blended Teaching Mode, Balint Group Teaching Mode, Micro video Teaching Mode, PBL Teaching Mode, Carolina Teaching Mode, and Step-by-Step Teaching Mode. Although the network meta-analysis provided insights into the effectiveness of these teaching modalities, it is crucial to consider their unique strengths and limitations. Therefore, our research team conducted a systematic review of these 12 modalities, focusing on four key aspects: enhancement of practical experience and emotional resonance, improvement of comprehensive abilities and flexibility, balancing systematic approach and interactivity, and consideration of resource requirements and adaptability. Additionally, it should also be noted that the ranking results analyzed in the meta-analysis of this study do not imply statistical significance between each pair of comparisons. What they show is the best probability result obtained in our probabilistic analysis.

### 4.1 Enhancement of practical experience and emotional resonance

The Peer Teaching Mode, Experiential Teaching Mode and Narrative Nursing Teaching Mode stood out in terms of practical experience and emotional resonance. Peer Teaching Mode (As shown in Figure 3, Reflective Mode yielded an SMD of 4.06 95% CI: 3.55–4.56, ranking mid-tier, SUCRA: 91.7%) involves matching new nurses with senior nurses based on personality traits, enabling personal guidance and demonstration. This approach helps new nurses quickly integrate into the clinical environment, master practical skills and humanistic care techniques, while receiving emotional support and encouragement. Moreover, compared to other modalities, the experiential and mentoring aspects of Peer Teaching Mode enhance new nurses’ self-confidence and professional identity, which could explain its superiority. The Experiential Teaching Mode (As shown in Figure 3, Reflective Mode yielded an SMD of 1.63 95% CI: 0.04–3.21, ranking mid-tier, SUCRA: 52.4%) allows students to experience nursing processes firsthand, such as role-playing as patients, deepening their understanding of patients’ needs and feelings, and fostering empathy. The Narrative Nursing Teaching Mode (As shown in Figure 3, Reflective Mode yielded an SMD of 2.05 95% CI: 1.30–2.80, ranking mid-tier, SUCRA: 61.1%) stimulates emotional resonance and cultivates humanistic care awareness by telling real-life nursing stories. However, these models have limitations, such as the dependence of Peer Teaching Mode on the individual abilities and teaching styles of mentors, the need for specific environments and resources in Experiential Teaching Mode, and the potential subjectivity in story selection affecting the consistency of teaching effectiveness in Narrative Nursing. Additionally, in the funnel chart presented in this study, we can see that the experiential teaching mode that lies outside the scope of the funnel chart has a higher risk of reporting bias. The experiential teaching mode is characterized by a relatively short training duration (≤3 months), which is generally shorter than that of other innovative teaching modes and is also quite different from the optimal training duration identified in our subgroup analysis (>6 months). Therefore, if we want to improve the impact of the experiential teaching mode on the humanistic care ability of nursing staff, future studies should consider extending the training duration of this teaching mode.

### 4.2 Improvement of comprehensive abilities and flexibility

PBL Teaching Mode and Blended Teaching Mode excel in enhancing comprehensive abilities and flexibility. PBL Teaching Mode (As shown in Figure 3, Reflective Mode yielded an SMD of 1.08 95% CI: 0.36–1.81, ranking mid-tier, SUCRA: 36.9%), a problem-oriented approach, involves group collaboration to solve practical problems, thereby improving students’ active learning, problem-solving skills, teamwork, and communication abilities. Blended Teaching Mode (As shown in Figure 3, Reflective Mode yielded an SMD of 1.59 95% CI: 0.91–2.28, ranking mid-tier, SUCRA: 50.1%) combines the advantages of online and offline teaching, allowing flexible adjustment of teaching methods based on educational objectives and student needs. For instance, theoretical knowledge can be imparted through online courses, while practical operations and discussions take place offline. However, these models have higher implementation costs, with PBL Teaching Mode requiring rich teaching resources and case support, and Blended Teaching Mode necessitating technical support and equipment investment.

### 4.3 Balancing systematic approach and interactivity

The traditional Teaching Mode and the ADDIE Teaching Mode each have their focus on systematic approach and interactivity. The traditional Teaching Mode primarily lecture-based, systematically imparts nursing knowledge and skills, ensuring students grasp fundamental concepts. However, it lacks student engagement and interaction, potentially affecting learning outcomes and emotional development. The ADDIE Teaching Mode (As shown in Figure 3, Reflective Mode yielded an SMD of 2.46 95% CI: 1.99–2.92 ranking mid-tier, SUCRA: 67.7%), consisting of analysis, design, development, implementation, and evaluation, ensures comprehensive and scientific teaching design. Its multi-step process and complexity require considerable time and resources. The Lasswell Teaching Mode (As shown in Figure 3, Reflective Mode yielded an SMD of 3.15 95% CI: 2.30–4.00, ranking mid-tier, SUCRA: 79.6%) provides a systematic framework for the training of nurses’ humanistic care abilities. This model consists of five elements: “Who”, “Says what”, “In which channel”, “To whom”, and “With what effect”. This framework facilitates the comprehensive design and evaluation of training outcomes. The training content based on the Lasswell model covers multiple modules, including humanistic care theory, communication skills, emotional management, and self-care. These modules aim to enhance nurses’ humanistic care abilities, enabling them to better understand and care for patients’ needs. Conversely, Reflective Teaching Mode (As shown in Figure 3, Reflective Mode yielded an SMD of 1.71 95% CI: 1.04–2.38, ranking mid-tier, SUCRA: 51.9%) and the Balint Group Teaching Mode (As shown in Figure 3, Reflective Mode yielded an SMD of 1.32 95% CI: 1.04–1.61, ranking mid-tier, SUCRA: 43.6%) emphasize interactivity and emotional support. Reflective Teaching Mode encourages self-reflection and summation, enhancing self-awareness and professional identity. The Balint Teaching Mode fosters emotional support and professional identity through group discussions and sharing, helping students navigate complex patient-provider relationships and emotional issues. These modes, however, demand significant time investment from students for reflection and discussion.

### 4.4 Consideration of resource requirements and adaptability

The Micro-video Teaching Mode (As shown in Figure 3, Reflective Mode yielded an SMD of 1.13 95% CI: 0.53–1.73, ranking mid-tier, SUCRA:38.6%) and the Carolina Teaching Mode (As shown in Figure 3, Reflective Mode yielded an SMD of 1.05 95% CI: 0.64–1.46, ranking mid-tier, SUCRA: 35.6%) have relatively higher resource requirements. Micro-video Teaching Mode involves creating concise and informative videos of teaching content, which are then disseminated and used for learning through the internet and mobile devices. Micro-video Teaching Mode necessitates professional equipment and techniques to produce high-quality videos, but its vivid presentation enhances student interest and comprehension. The Carolina Teaching Mode emphasizes practical operations and clinical applications, demanding substantial practical resources and equipment. This model is originally applied more frequently in educational institutions such as North Carolina State University. While the Step-by-Step Teaching Mode (As shown in Figure 3, Reflective Mode yielded an SMD of 0.87 95% CI: 0.26–1.48, ranking mid-tier, SUCRA: 33.4%) performs well in gradual advancement and systematic approach, its fixed teaching content and steps can be challenging to adapt to varying educational needs and individual differences.

The practical process of this study adhered to the reporting requirements of network meta-analysis. No inconsistencies were observed among the included studies. However, there are limitations to consider: (1) The methodological quality of the included studies was moderate, with multiple unclear risk of bias assessments. Only 17 studies reported specific methods for random sequence generation, and six mentioned allocation concealment but did not specify the type of blinding used. This could introduce selection bias, implementation bias, and measurement bias, increasing heterogeneity among Meta-analysis studies. (2) Although we searched relevant databases both domestically and internationally, most included studies originated from China. No eligible foreign studies were identified based on our inclusion and exclusion criteria. This may be due to the study’s high standards for experimental design and thematic requirements, limiting the scope of inclusion to RCTs related to teaching modalities. (3) Many RCTs did not mention the teaching resources and training details required for this innovative teaching model, and thus, further subgroup analyses could not be performed. Future research should aim to directly compare different teaching modalities to overcome the limitations of indirect comparisons and validate the findings of this study. (4) Due to the lack of uniform outcome indicators for assessing many innovative models and the limited number of RCTs (Randomized Controlled Trials) for the same innovative model, it is not feasible to conduct a proper direct comparison of the effectiveness between innovative models. (5) Most of the RCTs we included failed to conduct a long-term follow-up on the humanistic care training outcomes of the participants, which prevents us from assessing and discussing the long-term impact of this teaching model. (6) Most of the RCTs failed to blind the participants or the assessors, so these studies may have a certain degree of selection bias (7) In the Network Structure, some models (e.g., Step-by-Step, ADDIE, Micro-video). had very thin links, indicating limited evidence.

### 4.5 Implications for nursing practice and future research

This network meta-analysis highlights the Peer Teaching Mode as the most effective approach to enhancing nurses’ humanistic care competencies, offering practical guidance for nursing education reform. Healthcare institutions are encouraged to adopt peer-assisted frameworks that promote both clinical integration and emotional support. While other models like ADDIE and Lasswell also show promising outcomes, their application requires significant institutional readiness and resource investment. The analysis further suggests that extended training durations, particularly those over 6 months, yield stronger and more lasting improvements. Future research should prioritize multi-center, international studies to validate these findings across cultural settings and explore hybrid models that integrate the strengths of various teaching strategies. Additionally, long-term follow-up is essential to assess whether gains in humanistic care persist and influence patient outcomes. Finally, developing standardized, multidimensional evaluation tools is critical for improving the consistency and comparability of future studies. Together, these recommendations can drive more effective training and advance humanistic nursing education globally.

## 5 Conclusion

In summary, all 12 novel teaching modalities effectively enhance nurses’ humanistic care abilities. However, there are variations in their effectiveness, Peer Teaching Mode was associated with the highest-ranking probability (91.7%) and the strongest pooled effect size (SMD = 4.06, 95% CI: 3.55–4.56), although confidence intervals suggest possible overlap with other effective modalities. Nevertheless, each individual teaching modality has its limitations and may not fully meet the demands of cultivating humanistic care abilities in nursing. Therefore, we suggest that future studies should use of multi-country datasets to generalize findings, exploration of hybrid teaching approaches (e.g., Peer + PBL) to combine strengths and measurement of long-term impact on clinical behavior or patient satisfaction.
